# Identifying Mechanisms of Action for Implementation Strategies Using a Retrospective Implementation Mapping Logic Model Approach

**DOI:** 10.1007/s11121-025-01790-2

**Published:** 2025-02-21

**Authors:** Timothy J. Walker, Funlola Are, Natalia I. Heredia, Kempson Onadeko, Emma E. Saving, Eunyoung Kang, Maria E. Fernandez

**Affiliations:** 1 Center for Health Promotion and Prevention Research, School of Public Health, University of Texas Health Science Center at Houston, Houston, TX USA; 2 Institute for Implementation Science, University of Texas Health Science Center at Houston, Houston, TX USA; 3 Louis A. Faillace, M.D., Department of Psychiatry and Behavioral Sciences, McGovern Medical School, University of Texas Health Science Center at Houston, Houston, TX USA

**Keywords:** Implementation, Implementation strategy, Logic model, Mechanisms, Implementation Mapping

## Abstract

Understanding causal mechanisms for implementation strategies is a priority for implementation and health promotion research. Logic models are helpful for understanding and illustrating mechanisms through which implementation strategies operate. Little guidance exists for developing logic models for existing implementation strategies. We demonstrate how to use Implementation Mapping (IM) in a retrospective manner to develop an IMap Logic Model for a social-emotional learning program implemented at Boys & Girls Clubs of Greater Houston (BGCGH). To inform logic model development, we used qualitative interview data (from implementers) and conversations with program organizers. To develop the logic model, we specified the most accessible information, beginning with the program and health-related outcomes. We then specified the implementation strategies and implementation outcomes, followed by change methods (i.e., theoretical techniques that influence positive change in determinants), practical applications (i.e., specific techniques for operationalizing change methods), and determinants (factors that influence implementation) and other contextual factors. The effectiveness outcomes for the program were to improve emotion regulation and social skills among youth. Clinician implementers delivered the program and BGCGH club directors and staff supported delivery. The implementation strategies were (1) group leader trainings; (2) implementation guide; (3) BGCGH staff training; (4) needs assessments (via site visits); (5) follow-up meetings; and (6) pilot program check-in meetings. Collectively, the strategies used various practical applications (e.g., scenario discussions, reviewing procedures) to operationalize change methods (e.g., active learning, participatory problem solving) to address determinants (e.g., knowledge, interorganizational relationships). The strategy set out to improve implementation behaviors (e.g., delivering program components as prescribed) and implementation outcomes (e.g., fidelity). The developed IMap Logic Model can be used to inform implementation evaluation efforts by helping identify outcomes, mediators, and moderators. The logic model can also be used to identify gaps that, if addressed, can help ongoing implementation and scale-up efforts.

## Introduction

Understanding how implementation strategies impact implementation and effectiveness outcomes is a priority for implementation and health promotion research (Lewis et al., 2018, 2020, 2024; Powell et al., 2019). Implementation strategies are methods or techniques used to enhance the adoption, implementation, and sustainability of a program or practice (Proctor et al., 2013; Fernandez et al., 2019; Powell et al., 2014). Given that program implementation occurs in real-world settings that are inherently complex, implementation strategies can take many different forms. For example, they can consist of a single action or process (e.g., ongoing training), or they can have multiple components used together (e.g., ongoing training with facilitation) (Kang & Foster, 2022). The variation among strategies and the complexity of settings can make it difficult to understand *how* strategies impact implementation outcomes (effects of purposive actions to implement new practices) (Proctor et al., 2011). Without more clarity about implementation strategy components and mechanisms (i.e., reasons why and how change occurred), researchers and practitioners will continue to face challenges when developing, selecting, adapting, and scaling strategies to achieve project goals and more broadly improve public health.

There are calls for action to improve tracking and reporting of implementation strategies, and specifying and testing their mechanisms (Lewis et al., 2018, 2020; Powell et al., 2019). Research to improve understanding of mechanisms highlights the need to better specify strategy components, establish theoretical links between strategies and mediators, identify the outcomes they impact, and further examine the unique contributions of strategy components (Lewis et al., 2020; Williams, 2016). One way to help address current research needs is through studies designed to quantitatively test multilevel mediators of implementation (Lewis et al., 2020). However, these studies need to be carefully designed and theoretically informed, which can present additional challenges in community settings where foundational implementation research is still emerging (Balis & Houghtaling, 2023). In addition to quantitative studies, qualitative approaches can be a helpful way to gain a theoretical understanding of causal linkages between implementation strategies, the determinants they address, and the outcomes they impact (Brewster et al., 2015; Lewis et al., 2020; Springer et al., 2021).

Conducting research to better understand implementation strategies in community settings is especially important given the reach, variety, and scalability of programming. For example, organizations such as Boys & Girls Clubs implement numerous programs to about 3.3 million youth in a typical year across more than 5200 sites (Boys & Girls Clubs of America, 2022). Their programming includes sports and recreation, education, arts, health and wellness, workforce readiness, and character and leadership. Boys & Girls Clubs use multiple implementation strategies to deliver programs to youth most in need (Boys & Girls Clubs, 2023). These strategies include staff trainings, organized program listings and resources, and reference manuals for sites and staff. However, understanding how existing implementation strategies used in community settings such as Boys & Girls Clubs can be challenging given the different strategy components, determinants they address, contextual factors involved, and outcomes targeted. A lack of understanding of how implementation strategies work can further impede efforts to scale-up effective programming. There are multiple factors that affect scale-up including a skilled workforce capable of delivering evidence-based interventions (EBIs) (Fagan et al., 2019). Thus, knowing how strategies build implementation skills to achieve desired outcomes is critical for sustained delivery of EBIs to produce population-wide improvements.

There are existing tools and approaches to help understand implementation strategies including Mechanism Mapping, Causal Pathway Diagraming, and the Implementation Research Logic Model (Geng et al., 2022; Kilbourne et al., 2023; Klasnja et al., 2024; Smith et al., 2020). These approaches are useful when describing mechanisms, although they lack step-by-step guidance and/or can be challenging to apply to multicomponent strategies that have mechanisms operating across levels (i.e., strategies that affect individual- and organizational-level determinants). Implementation Mapping (IM) can help inform research designed to understand existing implementation strategies, and can be especially helpful in understanding mechanisms of multicomponent strategies (Fernandez et al., 2019). IM is traditionally used in a prospective manner to develop, select, and tailor implementation strategies. The IM process consists of five tasks that begin with a needs/assets assessment and end with evaluation planning (Table 1). Through the process, users identify *who* is involved in implementation, *what* implementation outcomes and behaviors need to be addressed, *why* implementation behaviors and outcomes are achieved (i.e., implementation determinants), and *how* to address implementation determinants (at the individual, organization, community, and other ecological levels). By working through the tasks, users develop implementation strategies that link outcomes to behaviors, behaviors to personal and contextual determinants, and determinants to strategies. This sequence of connections can be further depicted using an Implementation Mapping (IMap) Logic Model, which helps to illustrate the core components and mechanisms of action for the strategy.

**Table 1:** Implementation Mapping (IM) tasks, purpose, and retrospective mapping

IM task	Purpose (*question addressed*)	Retrospective mapping
1. Conduct needs and assets assessment and identify adopters and implementers	Understanding needs & assets (*Who?*)	Identify the program and the adoption and implementation roles (1)
2. Identify adoption and implementation outcomes, performance objectives, and determinants; create matrices of change	Planning change (*What and Why?*)	Identify implementation outcomes and implementation behaviors (2) Identify determinants (5)
3. Choose theoretical change methods; select or create implementation strategies	Choosing & operationalizing implementation strategies (*How?*)	Identify existing strategies (3), practical applications, and change methods (4)
4. Produce implementation protocols and materials		
5. Evaluate implementation outcomes	Evaluating (*Is it working?*)	Identify potential gaps in an existing strategy (6)

The number in the “Retrospective mapping” column represents the order in which the information was specified during the mapping process

Key features of IM are the structured tasks and resources that help users to systematically link outcomes to strategies (Kok et al., 2016) (Eldredge et al., 2016). IM can also be used to understand and enhance existing strategies (Schultes et al., 2022). More specifically, IM principles can be used to systematically “map” existing strategies by identifying the strategy components and linking them to implementation outcomes through the use of an IMap Logic Model (Table 1). The information provided in the IMap Logic Model can be valuable when improving ongoing implementation efforts and expanding efforts to support program scale-up. Despite the promise, there is a lack of guidance and examples of using IM and IMap Logic Models to “map” existing strategies. Therefore, the purpose of this study is to provide a descriptive documentation of a methodology for understanding mechanisms of existing implementation strategies. The study showcases how to “map” an existing strategy from a real-world setting using IM and an IMap Logic Model. The strategy in this example is designed to support the delivery of Strong Kids, a social-emotional learning program implemented at Boys & Girls Clubs of Greater Houston (BGCGH).

## Methods

### Partnership Background

In 2021, the Boys & Girls Clubs of Greater Houston (BGCGH) partnered with faculty at UTHealth Houston McGovern Medical School (referred hereafter as the program team) to pilot Strong Kids, a social-emotional learning program for youth. Studies indicate Strong Kids has increased student’s knowledge of healthy social-emotional behavior, reduced internalizing problem symptoms, increased social-emotional competencies and resilience, and decreased problem behavior (Merrell, 2010; Merrell et al., 2008, Harlacher & Merrell, 2010). In collaboration with the program team (which consisted of faculty and postdocs), BGCGH implemented the program at 7 of 23 sites from 2021 to 2022. An internal evaluation conducted by the program team revealed there was evidence that the program improved self-awareness, self-management, social-awareness, relationship skills, responsible decision making, personal responsibility, and optimistic thinking among youth at pilot sites.

In 2022, UTHealth Houston School of Public Health (SPH) faculty initiated a meeting with BGCGH leadership about ongoing programs and implementation priorities. BGCGH leadership expressed an interest in scaling up the Strong Kids program and a desire to better understand implementation challenges as part of the scale-up process. In response, the UTHealth Houston SPH team (referred hereafter as the UTHealth Houston implementation research team) and BGCGH successfully obtained a Community Health Initiated Research Partnership award that focused on partnership building and research to support implementation and scale-up planning. The partnership building process included a full-day retreat to launch the partnership and monthly meetings to collaboratively execute partner-driven research. In 2022–2023, the UTHealth Houston implementation research team (consisting of faculty, a doctoral student, and a master’s student) initiated research activities by first conducting an implementation needs and assets assessment for the Strong Kids program. The primary goals of the assessment were to (1) identify program adopters and implementers, and their implementation roles; (2) gain an understanding of the current implementation strategies used at BGCGH and specifically for the Strong Kids program; and (3) identify barriers/facilitators to program implementation for Strong Kids, and programming in general. The Committee for the Protection of Human Subjects at The University of Texas Health Science Center at Houston approved the study.

### Needs and Assets Assessment

The implementation research team used an exploratory sequential mixed methods design, which included a qualitative phase followed by a quantitative phase to conduct the overall needs and assets assessment. This study specifically uses qualitative data collected from the needs assessment to create the IMap Logic Model to understand implementation strategy mechanisms. The assessment was informed by the Interactive Systems Framework for Dissemination and Implementation Research (ISF), the R = MC^2^ heuristic (Readiness = Motivation × Innovation Specific Capacity × General Capacity), and the Implementation Mapping process (i.e., identifying specific adopters and implementers, their implementation behaviors, and determinants of their behaviors) (Fernandez et al., 2019; Scaccia et al., 2015; Wandersman et al., 2008). The ISF and R = MC^2^ heuristic helped guide study planning and the interpretation of qualitative results. Specifically, the ISF describes three systems, which helped inform who from these systems the team approached for interviews. The IM process helped structure the interview guide by including questions about *who* was involved with implementation, *what* they needed to do and why (i.e., determinants), and *how* current efforts addressed determinants. The R = MC^2^ heuristic supported the identification of implementation determinants as described in the “Analysis” section.

For the qualitative phase, the implementation research team worked with the program team leader and BGCGH leadership to develop the interview protocol including the recruitment approach, data collection procedures, and interview guide. The implementation team conducted interviews with (1) Group Leaders of the Strong Kids program (postdocs from UTHealth Houston program team); (2) BGCGH club directors; and (3) staff leads and youth development specialists from sites that used the Strong Kids program. We chose to interview these roles given they represented people from the delivery system of the ISF and they could speak to different aspects of Strong Kids implementation.

### Qualitative Recruitment, Data Collection, and Analysis

#### Recruitment

The implementation research team used a purposive sampling approach to recruit Group Leaders, Club Directors, and staff leads or youth development specialist(s) who supported the Strong Kids program at respective sites. Leaders from the UTHealth Houston program team provided contact information for Group Leaders, and BGCGH leaders provided contact information for BGCGH Club Directors and staff at sites that used the Strong Kids program. Members of the UTHealth Houston implementation research team recruited interview participants. Specifically, implementation research team members emailed and/or called potential participants to explain the project, discuss their interest, and arrange an interview time. Two members of the implementation research team conducted all interviews.

In total, there were seven pilot sites that implemented the Strong Kids program. There were two Group Leaders who led Strong Kids groups at the pilot sites. Within each site, there was a club director and about 1–2 staff members that supported the Strong Kids program at a respective site. The implementation team completed 12 individual interviews in total with Group Leaders (*n* = 2), BGCGH club directors (*n* = 7), and BGCGH support staff (*n* = 3) at sites using the Strong Kids program. Notably, there was at least one participant interviewed from each pilot site. There were fewer support staff represented because of staff turnover between the time a site ended the program and time of interviews. The majority of participants were female (*n* = 9) and the most represented age category was 26–35 (*n* = 5). Club directors had an average of 2.5 years in their positions, support staff had 5 years, and Group Leaders had 3 years.

#### Data Collection

Researchers from the UTHealth Houston implementation research team completed both in-person and virtual semi-structured individual interviews in the fall of 2022 and winter of 2023. The interviews lasted about 45–60 minutes and interviewees received a $30 gift card for their participation. For in-person interviews, participants provided written consent, and for virtual interviews, participants waived documentation of written consent and gave verbal consent. Researchers recorded interviews and sent recordings of in-person interviews to an automated transcription company or used transcriptions provided by the virtual platform (for virtual interviews). The implementation team reviewed and cleaned all transcriptions. Researchers used a semi-structured interview guide that included questions and probes about implementation roles, barriers/facilitators to implementation, and implementation strategies. The implementation team determined the final sample size by ensuring there was at least one participant from each pilot site, the richness of the data, and whether additional participants were providing new information (Moser & Korstjens, 2018).

#### Analysis

The UTHealth Houston implementation research team used Rapid Assessment Procedures to analyze qualitative findings (Hamilton & Finley, 2019; Hamilton, 2013; Kowalski et al., 2024). Specifically, the team created written interview summaries from each interview audio file using a summary template in Microsoft Word. The template included domains informed by interview guide questions and bulleted statements from interviews captured the key information related to the domain. The team then created matrices for interviews using Microsoft Excel that included participant numbers down each row and domains informed by the interview guide questions across the columns. The team transferred content from interview summaries to matrices, which were then used to identify and synthesize information across interviews. Specifically, the team used the matrices to synthesize information about (1) the program and implementer roles, (2) implementation behaviors and outcomes, (3) implementation strategies, (4) change methods and practical applications used within strategies, and (5) implementation determinants (i.e., barriers/facilitators). This deductive approach allowed for a structured analysis to capture the relevant information to create an IMap Logic Model, which was the primary aim of this study.

To synchronize our findings with the often-used compilation of implementation strategies referred to as the Expert Recommendations for Implementation Change (ERIC) strategies, our team aligned the described implementation strategies with ERIC and ERIC SISTER (School Implementation Strategies, Translating ERIC Resources) strategies (when possible) (Cook et al., 2019; Powell et al., 2015). We also used R = MC^2^ to help identify and organize the discussed determinants of implementation (Scaccia et al., 2015). For example, when participants described a strategy component, the respective component was linked to a corresponding ERIC SISTER strategy (e.g., conduct ongoing training, conduct local needs assessment). Additionally, when participants described barriers/facilitators to implementation, the analytic team aligned them with existing constructs from the R = MC^2^ heuristic (e.g., needing to work well with other organizations was aligned with interorganizational relationships). The UTHealth Houston implementation research team shared and discussed the tables with the program team leader and BGCGH leadership to obtain additional feedback and finalize the content.

The team also used the qualitative data and conversations with the program leader and BGCGH leaders to further describe implementation strategy components consistent with current reporting recommendations (Proctor et al., 2013). These components include specifying the actors (who enacts the strategy); the action (actions that need to be enacted); action target (targets according to conceptual models of implementation); temporality (when the strategy is used); dose (dosage of implementation strategy); implementation outcome affected (the implementation outcome likely affected by each strategy); and justification (empirical, theoretical, or pragmatic justification). Specifying the strategy components are meant to complement the information in the IMap Logic Model.

### Developing the IMap Logic Model

The UTHealth Houston implementation research team co-developed the IMap Logic Model with project partners. Traditionally, the IMap Logic Model is developed prospectively and in a right-to-left manner, meaning a user begins with outcomes and works backward towards the strategy. From a logic standpoint, the IMap Logic Model is read left-to-right beginning with the evidence-based intervention. Given we used a retrospective development approach, we specified the logic model components based on the most accessible information first, which meant working from the outside-in: (1) identifying the program and effectiveness outcomes (IM Task 1); (2) specifying implementation outcomes and behaviors (IM Task 2); (3) listing implementation strategies (IM Task 3); (4) identifying change methods and practical applications (IM Task 3); and (5) and specifying determinants and other contextual factors (IM Task 2) (Table 1).

**Fig. 1:**
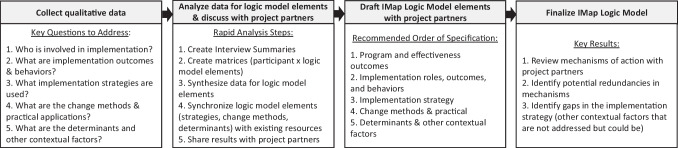
Overview of the retrospective IMap Logic Model development approach

The team leveraged existing resources, theories, and frameworks to help specify implementation strategies, outcomes, change methods, determinants, and other contextual factors (Powell et al., 2015) (Proctor et al., 2011) (Kok et al., 2016) (Scaccia et al., 2015). Table 2 includes definitions of the logic model components and sample questions from the interviews that generated information about respective components. During the logic model development process, the UTHealth Houston implementation research team worked with partners to draft elements, fill in gaps, and finalize content. For example, after completing interviews, the implementation team held virtual meetings with the program leader and BGCGH leaders to share descriptions of implementation roles, barriers/facilitators, and implementation strategy components generated from interview data. The meetings also included discussions about information gaps such as specific resources used in training sessions (e.g., PowerPoint slides) and confirming different training formats (e.g., in-person vs. virtual). Figure 1 provides an overview of the IMap Logic Model development approach. When developing the IMap Logic Model, the team first specified the program and effectiveness outcomes followed by the rest of the IMap Logic Model in the following order: (1) implementation roles, outcomes, and behaviors; (2) implementation strategy components; (3) change methods (theoretical techniques that influence positive change in determinants) and practical applications (specific techniques for operationalizing change methods); (4) determinants (factors that influence implementation) and other contextual factors (i.e., factors that influence implementation but are not directly impacted by the implementation strategy).

**Table 2:** Implementation Mapping Logic Model components and corresponding interview questions

Logic model component	Definition	Interview questions^a^
Implementation outcomes	Effects of purposive actions to implement a program	What was the goal of the [implementation strategy]? What is your role in delivering [the program]? Who else was involved in delivering [the program]?
Implementation behaviors	Tasks an implementer needs to carry out in order to achieve implementation outcomes	
Determinants	Factors that influence implementation behaviors (both at individual and higher levels)	What makes it difficult (or easy) for you to deliver [the program]? What challenges does your site face when delivering [the program]?
Change methods	Theoretical techniques that influence positive change in determinants of implementation behaviors	What was the format [the training]? What resources were used for [the training]? What activities did you do during [the training]?
Practical applications	Specific techniques for operationalizing a change method	
Implementation strategies	Methods or techniques to enhance implementation	What did you or your organization do to prepare for delivering the [program]? What was most helpful to support [program] delivery? What strategies did you use to support [program] implementation?
Other contextual factors	Factors that may influence implementation but they are not directly impacted by the implementation strategy	What makes it difficult (or easy) for you to deliver the [program]? What challenges does your site face when delivering the [program]? What was most helpful to support [program] delivery?

^a^Interview questions are not listed in order, and generally followed this sequence: (1) questions about the program, (2) implementation roles, (3) what makes implementation easy/difficult, (4) what did your organization do to prepare for implementation, (5) format/resources/activities/goals of implementation strategies discussed

## Results

### Program Description and Effectiveness Outcomes

The effectiveness outcomes for the Strong Kids program are improving emotion regulation and building social skills among youth with internalizing behaviors. The UTHealth Houston program team and BGCGH adapted the program from *Merrell’s Strong Start* and *Strong Kids* curricula to consist of six (45–60 min) sessions delivered during out-of-school time hours (i.e., after school or during the summer) (Whitcomb & Merrell, 2012) (Carrizales-Engelmann et al., 2016a) (Carrizales-Engelmann et al., 2016b). Sessions took place at the clubs and followed a standard pre-determined format, although group leaders often tailored examples to scenarios or situations that BGCGH staff deemed relevant for the youth participating. For youth in grades K-2, the sessions consisted of the following: (1) Intro to Group and Understanding Feelings 1; (2) Understanding Feelings 2 & Understanding Others’ Feelings; (3) What to Do When You Are Angry; (4) What to Do When You Are Happy; (5) What to Do When You Are Worried; and (6) Being a Good Friend/Solving People Problems. For youth in grades 3–5 and 6–8 groups, sessions consisted of the following: (1) Intro to Group and Understanding Emotions 1; (2) Understanding Emotions 2 & Understanding Others’ Emotions; (3) Dealing with Anger; (4) Clear Thinking 1 & 2; (5) Solving People Problems; and (6) Letting Go of Stress/Positive Living.

BGCGH sites organized multiple groups based on the age of youth members eligible and interested in the program (e.g., k-2nd grade, 3rd–5th grade, 6th–8th grade). Groups generally consisted of 5 to 10 youth members depending on the size of the club and number of youths deemed eligible for the program. Overall, BGCGH sites screened youth members for eligibility, enrolled youth in the program, and delivered Strong Kids sessions.

### Implementation Roles, Outcomes, and Behaviors

Interview participants described the different implementation roles and responsibilities. More specifically, the Group Leaders were psychologists in postdoctoral positions at UTHealth Houston. Group Leaders had multiple implementation behaviors including implementing all six program lessons to youth members and delivering the core components of each lesson (Table 3). Given these behaviors, the corresponding implementation outcome was determined to be fidelity of Strong Kids delivery. BGCGH staff members also had multiple implementation behaviors including completing youth screening forms, being present during each lesson, and helping to manage youth behavior (Table 3). Thus, BGCGH staff implementation outcomes were specified as screening eligible youth and support program delivery during sessions. Implementation behaviors for club directors included working with staff to screen youth, speaking with parents about the program, designating a space for lessons, scheduling, and ensuring all necessary resources were available. Thus, their implementation outcomes were to screen eligible youth and support program implementation (Table 3). In total, 298 youth members were screened across the seven sites, using a program-specific screener that was developed by UTHealth program staff and BGCGH leadership that focused on social-emotional competencies using a CASEL framework (Payton et al., 2000).

**Table 3:** Implementation roles, behaviors, and outcomes

Position title & role	Implementation behaviors	Implementation outcomes
Group Leaders: primary implementer	- Provide guidance to club directors & staff about screening youth members - Review screening forms from BGCGH sites to identify eligible youth members - Implement all 6 lessons of the program to eligible youth members - Cover the most important components of each lesson	Implementation fidelity
BGCGH staff members: implementation support	- Support club directors in completing youth screening forms - Speak with parents about the program and obtain consent - Gather youth members at the start of each lesson - Be present during each lesson - Manage challenging youth behavior & foster youth engagement	Screen eligible youth Support program delivery during sessions
BGCGH Club Directors: implementation support	- Work with staff member(s) to complete youth screening forms - Speak with parents about the program and obtain consents - Designate a space at the club for the program to operate - Create a schedule for lessons - Ensure implementers and staff have necessary resources	Screen eligible youth Support program implementation

### Implementation Strategy

When asked about what their organization did to prepare for program delivery, participants discussed multiple strategies. These strategies included (1) group leader trainings; (2) implementation guide for group leaders; (3) trainings for BGCGH club directors and staff; (4) needs assessments (via site visits conducted by UTHealth Houston Group Leaders to understand program needs and meet BGCGH staff); (5) follow-up meetings about program implementation; and (6) pilot program check-in meetings after completing stages of implementation (e.g., meetings after recruitment process, meetings after initial sessions). Details about each respective strategy component are provided in Table 4, which is formatted based on current recommendations for specifying and reporting implementation strategies (Proctor et al., 2013).

**Table 4:** Implementation strategy component specification

	Group leader training	Implementation guide	BGCGH training	Needs assessment/site visits	Follow-up meetings	Pilot program check-in/check-out meetings
Actors	UTHealth Houston Program Organizer	UTHealth Houston Program Organizer/Group Leaders	UTHealth Houston Program Organizer	Group Leaders BGCGH Staff	UTHealth Houston and BGCGH Leaders	UTHealth Houston and BGCGH Leaders
Actions	Introduce group leaders to Strong Kids program & materials Discuss scenarios Demonstrate implementation	Provide group leaders with resources to launch and implement the program	Introduce BGCGH staff to the program and their implementation roles	Introduce Group Leaders to BGCGH staff Observe current programming Discuss current programs Designate room for program	Discuss programming needs Review & discuss procedures Provide feedback	Discuss programing needs Provide feedback
Action target	Group Leaders	Group Leaders	BGCGH Staff	Group Leaders & BGCGH Staff	UTHealth Houston Program Organizer Group Leaders BGCGH Leaders BGCGH Staff	UTHealth Houston Program Organizer Group Leaders BGCGH Leaders BGCGH Staff
Temporality	Before working with BGCGH sites	Before working with BGCGH sites*	Prior to program implementation	Prior to program implementation	After initial trainings and needs assessment, before implementation	Check-in after 2 weeks of group sessions Check-out after final group session
Dose	1 in-person session (2 h)	For review as needed	1 in-person session (2 h)	1 visit/site (1.5 h)	1 session (45 min–1 h)	1 session (1 h)
Implementation outcomes	Fidelity	Fidelity	Acceptability/fidelity	Interorganizational relationships	Fidelity Leadership support	Fidelity Leadership support
Justification	Improve knowledge Build skills	Improve knowledge Reduce barriers	Improve knowledge Improve self-efficacy	Understand site and expectations, build relationships	Address barriers	Address barriers

*The guide was developed after the first sites implemented the program and further refined throughout the implementation effort

### Practical Applications/Change Methods

The implementation strategies used practical applications and corresponding change methods. For example, the Group Leader trainings (implementation strategy) used PowerPoint slides and information sheets (practical applications) to organize information (advance organizers) to facilitate learning. The training sessions also included question and answer (Q&A) sessions and scenario discussions (practical applications) to engage Group Leaders in active learning and discussion (change methods). Lastly, the training included demonstrations of Strong Kids program components (practical application), which is a form of modeling (change method).

Similarly, the pilot program check-in meetings (implementation strategy) included Q&A sessions and discussions about program progress (practical applications) to engage UTHealth Houston Program organizers, Group Leaders, BGCGH leaders, and Club Directors in active learning and discussion (change methods). During the follow-up meetings, the team also reviewed procedures to identify potential problems and discuss solutions (practical application), which was a form of participatory problem solving (change method). Group leaders also completed needs assessments through site visits. During the visits, Group Leaders spoke (practical application) with BGCGH staff about implementation, which is a form of sense-making (change method).

A complete list of implementation strategies, practical applications, change methods, and determinants are provided in Table 5. The implementation strategy components and many of the practical applications emerged from the interview data directly. Some practical applications were clarified through conversations with the program leader and BGCGH leaders. Through further discussions with the program lead and BGCGH leaders, the implementation team aligned the change methods with the practical applications and strategy components.

**Table 5:** Implementation strategies, practical applications, change methods, and determinants

Implementation strategy	Practical applications	Change method	Determinants
Group leader training	PowerPoint lectures	Advance organizers	Knowledge Skills & self-efficacy
	Information sheets		
	Q&A sessions	Active learning	
	Scenario discussion	Discussion	
	Demonstration sessions	Modeling	
Implementation guide (for group leaders)	List of resources	Advance organizers Facilitation Technical assistance	Knowledge Overcoming barriers
	Order of operations		
	Tips & tricks sheet		
	Drafted communication materials		
BGCGH staff training	PowerPoint slides	Advance organizers Verbal persuasion	Knowledge, attitudes, self-efficacy, overcoming barriers
	Q&A sessions	Active learning	
	Program discussions	Discussion	
Needs assessments (via site visits)	Field observations	Direct experience	Outcome expectations Inter-organizational relationships
	Conversations with staff	Sense-making	
Follow-up meetings	Program discussions	Discussion Participatory problem solving	Knowledge, leadership support Overcoming barriers, leadership support
	Review needs assessment results		
	Review procedures		
Pilot program and check-in meetings	Program discussion	Discussion Participatory problem solving	Knowledge, overcoming barriers, inter-organizational relationships, leadership support
	Q&A	Active learning	
	Programmatic feedback	Feedback	

### Determinants

After identifying practical applications and change methods, the team linked them to corresponding determinants based on existing theoretical linkages highlighted in the taxonomy of behavior change methods, qualitative findings, and conversations with UTHealth Houston Program leaders (Kok et al., 2016). The taxonomy of behavior change methods represents a widely used resource that is central to the Intervention Mapping protocol. It includes theoretically based change methods along with corresponding definitions, parameters of use, and theoretical origins, and the determinants that these change methods are designed to influence. Thus, the taxonomy provides a consistent and systematic way to link strategy methods to specific determinants. For example, in the Group Leader trainings, the PowerPoint slides (practical application) that used advance organizers (change method) were thought to address Group Leader knowledge (personal determinant). This is because advance organizers is a change method linked to improving knowledge. Similarly, the session demonstrations (practical application) that operationalized modeling (change method) were thought to improve Group Leader skills/self-efficacy (personal determinant) because modeling is an approach to build self-efficacy (McAlister et al., 2008).

The Pilot program check-in meetings, which included program discussion, Q&A sessions, and programmatic feedback (practical applications), leveraged discussion, participatory problem solving, active learning, and feedback (change methods) to improve knowledge, overcoming barriers, interorganizational relationships, and leadership support (determinants). In the check-in meetings, program leaders, BGCGH leaders, Group Leaders, and BGCGH staff worked together to review progress and discussed what was working well and ways to address barriers. As a result, leaders were engaged in the process and could provide support to Group Leaders and support staff to improve implementation success. Similarly, the site visits, which included conversations between Group Leaders and BGCGH staff (practical application), leveraged sense-making (change method), and further improved inter-organizational relationships due to in-person interactions and co-planning. Table 5 also includes the personal and external determinants that were thought to be addressed by each implementation strategy.

### Other Contextual Factors

**Fig. 2:**
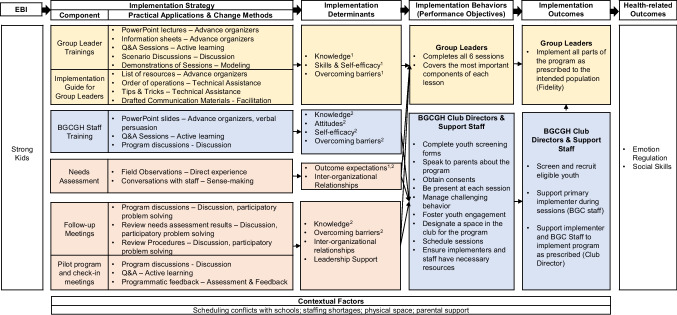
Strong Kids IMap Logic Model. Figure note: 1 refers to group leaders; 2 to Club Directors and Staff; yellow highlighting aligns mechanisms for Group Leaders; blue highlighting aligns mechanisms for BGCGH Club Directors & Support Staff; orange highlighting aligns mechanisms for all implementers

Interview participants discussed numerous determinants of implementation. Some determinants were directly addressed by implementation strategies (i.e., leadership support), whereas others were not. Thus, the team specified the unaddressed determinants as other contextual factors. These included youth interest in the program, scheduling conflicts with schools, staff shortages, physical space, and parental support. Figure 2 presents a complete IMap Logic Model for the implementation strategy. The Logic Model highlights all components along with the mechanisms of action through which the implementation strategies operate and other contextual factors that may serve as moderators.

## Discussion

Our work showcases how to retrospectively develop an IMap Logic model for an existing implementation strategy using a qualitative approach. The retrospectively developed IMap Logic Model can help illustrate the core components and mechanisms of action for existing implementation strategies, which is valuable for understanding how they work. The IMap Logic Model is also useful for identifying ways to improve current implementation and scale-up efforts because it can help to identify gaps and redundancies within a strategy. Lastly, this retrospective approach to develop an IMap Logic Model represents a valuable way to understand existing, real-world strategies used in diverse settings.

Specific to the purpose of this study, the reported IMap Logic Model helps to highlight key features of the Strong Kids implementation strategy, including the mechanisms of actions. For example, the group leader trainings and implementation guide (strategy components) leveraged information sheets and resource lists (practical applications) to operationalize advance organizers and facilitation (change methods) to improve knowledge and reduce barriers of group leaders (personal determinants). Similarly, the pilot program check-in meetings (strategy component) used discussions and feedback (practical applications and change methods) to improve knowledge and reduce barriers (personal determinants) as well as improve interorganizational relationships and leadership support (higher level determinants). The IMap Logic model helps to further illustrate how all strategy components address determinants through their change methods and practical applications. Collectively, improving personal and higher-level determinants can help ensure people carry out their implementation behaviors and achieve implementation outcomes.

The IMap Logic Model also helps to highlight ways to potentially improve the implementation strategy. For example, youth interest in the program, scheduling conflicts with schools, staff shortages, physical space, and parental support were all reported barriers to implementation by interview participants. However, these factors were not directly addressed by the existing implementation strategy. Thus, the existing strategy could be improved by enhancing or adding strategy components. More specifically, BGCGH club directors and staff could improve youth interest by strategically advertising the program in a way that is appealing for youth members. Additionally, BGCGH leaders could address school conflicts by proactively coordinating with schools or providing schedule guidance for club directors to minimize conflicts with afterschool tutorials or sports. To address staffing shortages, BGCGH and UTHealth Houston could expand their partnership by creating additional implementation support through student training opportunities (e.g., practicums). To address space issues, BGCGH leaders could provide guidance to club directors about the importance of holding group sessions in spaces free from distraction and with set-up materials. Lastly, to improve parent support, sites could use flyers to distribute to parents providing more information about programming, and BGCGH program leaders could include a training component for club directors and staff for how best to speak with parents about the program to encourage engagement.

This knowledge is also valuable for scale-up, which entails taking the program to new club sites. The IMap Logic Model allows for a holistic view of the implementation strategy, determinants, specific implementation behaviors, and outcomes, which can help ensure each new site’s context specific needs are addressed through the strategy. For example, scaling up a program across BGCGH would require implementing at sites that share space with schools. Based on information from the needs and assets assessment, shared space sites can have additional challenges related to room availability and goal coordination with school partners. The IMap Logic Model can help planners identify whether they can integrate additional support into existing strategy components or whether they need new strategy components to address the specific barriers at shared space sites.

There is a need to gain a better understanding of implementation strategies and their mechanisms (Powell et al., 2019) (Lewis et al., 2018) (Lewis et al., 2020) (Williams, 2016). This is especially true for community settings as most implementation research has been conducted in clinical settings (Balis & Houghtaling, 2023). Currently, knowledge gaps remain for understanding why implementation strategies are effective, and how best to link strategies to barriers/facilitators (i.e., determinants) both in clinical and community settings (Balis & Houghtaling, 2023; Waltz et al., 2019). Resources such as determinant frameworks (e.g., R = MC^2^, Consolidated Framework for Implementation Research), the ERIC, and ERIC SISTER strategies are valuable as they provide potential determinants and implementation strategies to consider (Cook et al., 2019; Damschroder et al., 2009; Powell et al., 2015; Scaccia et al., 2015). Notably, the implementation strategy components identified in the Strong Kids IMap Logic Model aligned with multiple ERIC SISTER strategies: conducting ongoing training, developing educational materials, facilitation/problem solving, conduct local needs assessment, conduct educational outreach visits, and stage implementation scale-up were reflected. However, aligning existing implementation strategy components with ERIC SISTER strategies is insufficient to understand why they work and how they influence determinants. ERIC strategies provide general guidance about types of approaches but not the details and mechanisms of strategies (including the specific content contained within them, the implementation determinants they are designed to address, and how they are operationalized). Therefore, approaches to design strategies (prospectively), and understand them (retrospectively) require processes such as IM and the IMap Logic Model.

Retrospectively developing an IMap Logic Model can help address gaps in understanding why and how existing strategies work. Notably, the IMap Logic Model helps to characterize each implementation strategy component based on their form (how it is delivered) and function (purpose for change) as this information is reflected in the change methods and practical applications. The form and function are critical features to identify for both complex health interventions and implementation strategies (Perez Jolles et al., 2019). Additionally, the IMap Logic Model connects the strategy components to the determinants they address and the subsequent implementation outcomes (by linking practical applications, change methods, determinants, behaviors, and outcomes). As a result, mechanisms of action are created through the process to showcase how strategy components can influence determinants and outcomes. Further, BGCGH has a wealth of experience adopting, implementing, and maintaining programs. Using a retrospective approach can provide an avenue to understand implementation practices in real-world settings such as BGCGH. This is especially valuable given the ERIC SISTER strategies were adapted by implementation researchers and lack input from practitioners and intermediaries (external organizations or individuals who support real world implementation) (Cook et al., 2019). Thus, gaining a better understanding of real-world strategies in community settings is critical for expanding our understand of the types of strategies used, their mechanisms of action, and impact.

### Strengths and Limitations

This is the first known study to showcase the retrospective development of an IMap Logic Model for an existing implementation strategy. We leveraged a collaborative qualitative approach, which included obtaining multiple perspectives from investigators and partners throughout the process to reduce bias and triangulate key findings (Richardson, 2005). Multiple researchers were involved with data collection and interpretation providing an additional form of investigator triangulation. We also shared findings with experts external to the research team to confirm the identified change methods were logical based on our data, which is consistent with an external audit (Richardson, 2005). We also leveraged existing implementation frameworks, resources, and the IM process. This work also demonstrates the feasibility of mapping existing implementation strategies and how the process can be used to enhance implementation and scale-up. Given that there are now existing step-by-step instructions for retrospectively developing an IMap Logic Model for existing strategies, this study is a critical first step in informing future research.

However, there are study limitations. We did not review implementation strategy materials such as PowerPoint slides or information sheets used for trainings. Thus, it is possible that some strategy components may not be impacting determinants as perceived through the interview results. Additionally, specifying practical applications, change methods, and determinants presented some challenges given the existing strategy was not originally developed by explicitly identifying these elements. As a result, some mechanisms were more implied (e.g., follow-up meetings engaged leaders, which in turn, was thought to improve leadership support), whereas other mechanisms were more explicit (e.g., follow-up meetings included discussions about the program, which is a known change method to improve knowledge). There is also not as much guidance for linking change methods to higher-level determinants compared to personal determinants, which represents an area of future work. Specific to the qualitative data collection, the team did not use a member checking process, which provides additional credibility to the data. The team also did not interview parents or youth, which could have provided valuable perspectives about the program and its implementation.

## Conclusions

There is an important need to understand how implementation strategies work. This is especially true for existing implementation strategies used in community settings. Our work showcases a way to retrospectively develop an IMap Logic Model using a qualitative approach. We demonstrate the feasibility of mapping the key strategy components, specifying mechanisms, and identifying ways to improve the strategy. The approach represents a novel way to document and learn from existing strategies to further improve implementation efforts in community settings.

## Data Availability

The data used and/or analyzed during the current study are available from the corresponding author on reasonable request.
